# Nigral proteasome inhibition in mice leads to motor and non-motor deficits and increased expression of Ser129 phosphorylated α-synuclein

**DOI:** 10.3389/fnbeh.2015.00068

**Published:** 2015-03-31

**Authors:** Eduard Bentea, Anke Van der Perren, Joeri Van Liefferinge, Anissa El Arfani, Giulia Albertini, Thomas Demuyser, Ellen Merckx, Yvette Michotte, Ilse Smolders, Veerle Baekelandt, Ann Massie

**Affiliations:** ^1^Department of Pharmaceutical Biotechnology and Molecular Biology, Center for Neurosciences, Vrije Universiteit BrusselBrussels, Belgium; ^2^Laboratory for Neurobiology and Gene Therapy, Department of Neurosciences, KU LeuvenLeuven, Belgium; ^3^Department of Pharmaceutical Chemistry and Drug Analysis, Center for Neurosciences, Vrije Universiteit BrusselBrussels, Belgium

**Keywords:** mouse model, lactacystin, non-motor behavior, Parkinson's disease, proteasome, α-synuclein

## Abstract

Parkinson's disease is a neurodegenerative disorder characterized by motor and non-motor disturbances. Various pathogenic pathways drive disease progression including oxidative stress, mitochondrial dysfunction, α-synuclein aggregation and impairment of protein degradation systems. Dysfunction of the ubiquitin-proteasome system in the substantia nigra of Parkinson's disease patients is believed to be one of the causes of protein aggregation and cell death associated with this disorder. Lactacystin, a potent inhibitor of the proteasome, was previously delivered to the nigrostriatal pathway of rodents to model nigrostriatal degeneration. Although lactacystin-treated animals develop parkinsonian motor impairment, it is currently unknown whether they also develop non-motor symptoms characteristic of this disorder. In order to further describe the proteasome inhibition model of Parkinson's disease, we characterized the unilateral lactacystin model, performed by stereotaxic injection of the toxin in the substantia nigra of mice. We studied the degree of neurodegeneration and the behavioral phenotype 1 and 3 weeks after lactacystin lesion both in terms of motor impairment, as well as non-motor symptoms. We report that unilateral administration of 3 μg lactacystin to the substantia nigra of mice leads to partial (~40%) dopaminergic cell loss and concurrent striatal dopamine depletion, accompanied by increased expression of Ser129-phosphorylated α-synuclein. Behavioral characterization of the model revealed parkinsonian motor impairment, as well as signs of non-motor disturbances resembling early stage Parkinson's disease including sensitive and somatosensory deficits, anxiety-like behavior, and perseverative behavior. The consistent finding of good face validity, together with relevant construct validity, warrant a further evaluation of proteasome inhibition models of Parkinson's disease in pre-clinical research and validation of therapeutic targets.

## Introduction

Parkinson's disease (PD) is an age-related neurodegenerative disorder affecting 1% of the world-wide population (Massano and Bhatia, 2012). The main symptoms of PD are motor-related, with the cardinal features of bradykinesia, motor rigidity, and rest tremor forming the basis of the neurological classification of this disorder. These typical motor disturbances are believed to be the manifestation of degeneration of dopamine (DA) producing neurons of the Substantia Nigra (SN) pars compacta (SNc) that project to the striatum. Furthermore, Lewy bodies, protein aggregates highly enriched in the presynaptic protein α-synuclein (Spillantini et al., 1997), are a pathological hallmark of this disorder, and it is currently believed that protein aggregation makes an important contribution to the progressive neurodegeneration observed in PD (McNaught and Olanow, 2006). Interestingly, ~90% of α-synuclein in Lewy bodies is phosphorylated at Ser129 (Fujiwara et al., 2002; Anderson et al., 2006), indicating a potentially important role of this post-translational modification in the formation of Lewy bodies and cell death processes observed in PD.

Although significant effort has been invested in developing strategies to stop disease progression, the current management of PD still relies on symptomatic treatment with DA replacement strategies. These approaches can be beneficial in the first years after diagnosis, however the relentlessly progressive nature of PD makes them less efficient in controlling the disease, and can be the cause of disabling drug-induced side-effects (Schapira, 2009). The failure to achieve neuroprotection in PD can stem from various causes, however one explanation for the inability to successfully translate positive pre-clinical findings could be the limited diversity of animal models used (Jenner, 2008).

Proteasomal dysfunction has recently emerged as potential contributor to cell death in PD (Lim, 2007; Ebrahimi-Fakhari et al., 2012). Post-mortem data obtained from sporadic PD patients demonstrate a decrease in all three catalytic activities of the 20S proteasome (McNaught and Jenner, 2001; McNaught et al., 2003; Tofaris et al., 2003), together with decreased expression of various 26S proteasome subunits (McNaught et al., 2002a; Grunblatt et al., 2004; Bukhatwa et al., 2010). Interestingly, these structural and functional defects in the 26/20S proteasomes seem to be specific for the SNc, and do not occur in other brain regions such as the striatum, or various cortical regions, where neuronal loss is not observed (Furukawa et al., 2002; McNaught et al., 2003; Tofaris et al., 2003). A dysfunctional proteasome system can lead to aberrant protein turnover, and build-up of damaged or misfolded proteins, and as such be one of the causes of the protein aggregation and neuronal death observed in PD (McNaught et al., 2001; Olanow and McNaught, 2006; Ebrahimi-Fakhari et al., 2012). Consistent with this hypothesis, local administration of lactacystin (LAC), a selective and potent proteasomal inhibitor, leads to nigrostriatal DA-ergic cell death when applied to the SNc of rats (McNaught et al., 2002b; Vernon et al., 2010; Mackey et al., 2013; Pienaar et al., 2015) or the medial forebrain bundle of mice (Xie et al., 2010). Furthermore, LAC models consistently demonstrate the presence of α-synuclein pathology (McNaught et al., 2002b; Fornai et al., 2003; Miwa et al., 2005; Vernon et al., 2010, 2011; Xie et al., 2010; Mackey et al., 2013; Pienaar et al., 2015), a feature difficult to reproduce in toxin-based models of sporadic PD.

Besides motor symptomatology, PD patients also present a series of non-motor symptoms, such as anxiety, depression, somatosensory dysfunction, or cognitive impairment (Chaudhuri and Schapira, 2009; Martinez-Martin and Damian, 2010; Conte et al., 2013) that have a significant impact on the quality of life (Martinez-Martin, 2011; Hinnell et al., 2012). Despite the important burden of non-motor symptoms in PD, they remain largely under-researched (Jain and Goldstein, 2012). While LAC-treated rats and mice develop parkinsonian motor impairment (Vernon et al., 2010, 2011; Xie et al., 2010; Mackey et al., 2013; Konieczny et al., 2014; Pienaar et al., 2015), it is currently unknown whether proteasomal inhibition models can be used to study non-motor symptoms in PD.

In the current study we further characterized the unilateral LAC model of PD, performed by stereotaxic injection of the toxin in the SNc of mice. We studied pathological changes by investigating the degree of nigrostriatal DA-ergic degeneration, as well as potential changes in α-synuclein and Ser129-phosphorylated (S129-P) α-synuclein expression, at 1 and 3 weeks post-lesion. Furthermore, we characterized the behavioral phenotype after LAC lesion both in terms of motor impairment, and non-motor symptoms, with specific emphasis on somatosensory dysfunction, anxiety- and depressive-like symptoms, and cognitive impairment.

## Materials and methods

### Animals

This study was performed on male C57BL/6J mice (Charles River Laboratories, France), 12-14 weeks of age at lesion. Mice were group-housed (2-6 mice per cage) under standardized conditions (25°C, 10/14 h dark/light cycle), with free access to food and water. Studies were performed according to national guidelines on animal experimentation and were approved by the Ethical Committee for Animal Experimentation of the Faculty of Medicine and Pharmacy of the Vrije Universiteit Brussel. The results are reported in accordance with the ARRIVE guidelines for reporting experiments involving animals (Kilkenny et al., 2010).

### Stereotaxic surgery

Mice were anesthetized with a mixture of ketamine (100 mg/kg i.p.; Ketamine 1000 Ceva, Ceva Sante Animale, Brussels, Belgium) and xylazine (10 mg/kg i.p.; Rompun 2%, Bayer N.V., Brussels, Belgium), and positioned in a Kopf Model 963 Ultra Precise Small Animal Stereotaxic Frame, with a mouse adaptor (David Kopf Instruments, California, USA). The skull was exposed, and a small hole was made through the skull above the left SNc. A volume of 1.5 μL LAC 2 μg/μL was injected into the left SNc at the following coordinates: AP -3.0, LM -1.0, DV -4.5 from bregma, according to the atlas of Paxinos and Franklin (Paxinos and Franklin, 2007). LAC solutions were prepared by dissolving 50 μg LAC (Cayman Chemicals, Michigan, USA) in 25 μL NaCl 0.9%. In order to minimize lesion variability due to degradation of the toxin *ex vivo* which can yield an inactive LAC analog (Dick et al., 1996), fresh LAC solutions were prepared for every four mice, and immediately stored on ice. Control sham-operated mice received the same volume of vehicle (NaCl 0.9%), at the same coordinates. To minimize unspecific tissue damage, microinjections were performed using a 10 μL Model 1701 RN Neuros Syringe (Hamilton Company, Reno, USA), at a flow rate of 0.5 μL/min. After injection, the syringe was left in place for an additional 5 min, and then slowly removed. At the end of the surgery, the skin was sutured, and mice received 4 mg/kg ketoprofen i.p. (Ketofen, Merial, Brussels, Belgium) for post-operative analgesia.

### Neurobehavioral assessment

At 1 and 3 weeks after LAC administration, mice were investigated in a variety of motor and non-motor based paradigms. Behavioral assessment was performed between 9:00 a.m. and 6:00 p.m. (during the light phase), with alternate testing of sham- and LAC-treated mice to ensure evaluation of both treatment groups during the same time of the day. For tests requiring real-time behavioral scoring (nest building test, adhesive removal test), blinding for treatment condition was ensured by the presence of an additional researcher unaware of the test order during evaluation. For tests integrated off-line (cylinder test, light/dark test, tail suspension test), the acquired video files were integrated in a blinded manner. For the remaining tests (rotarod, and open field tests), blinding for treatment was ensured by employing objective and automated integration systems (TSE RotarRod Systems, and Noldus Ethovision respectively). For each behavioral test, mice were acclimatized to the testing room at least 1 h prior to assessment.

### Rotarod test

Motor function was investigated using an accelerating rotarod system (TSE RotaRod Advanced, TSE Systems). Prior to surgery, mice were trained for 5 min at a constant speed of 5 rpm. During this initial training phase, mice were placed immediately back on the rod after falling, allowing them to get familiarized to the test. In the second phase of training, mice underwent 3 repeated trials of 1 min at a fixed speed of 5 rpm, with 3 min of rest between trials. For testing the rotarod performance at baseline and after lesion, mice underwent 5 repeated trials that started at constant speed of 5 rpm for 30 s, and continued with a 5–25 rpm accelerating protocol during 200 s, leading to a maximum total rod time of 230 s. Mice were allowed 3 min of rest between trials. The mean of the 5 test trials underwent statistical analysis.

### Cylinder test

Forelimb akinesia and somatosensory asymmetry were investigated in the cylinder test. In this behavioral paradigm, mice are placed in a glass cylinder (10 cm diameter, and 21 cm height), with their motor activity video-recorded during 3 min. The cylinder was placed next to 3 mirrors to allow visualization of the movement from all angles. Subsequent analysis of the recordings reveals asymmetry in forelimb use upon performing weight-bearing supporting contacts with the cylinder walls. We also evaluated thigmotactic scanning behavior, by calculating the amount of time spent scanning the cylinder walls with the ipsilateral side of the body compared to the contralateral side. Scanning behavior was considered when the mouse was actively exploring the cylinder wall, sniffing or performing head elongations in the direction of the wall.

### Adhesive removal test

Sensorimotor performance was assessed using the adhesive removal test, as described previously (Bouet et al., 2009). After mice were habituated to a transparent test box for 60 s, small adhesive strips (0.3 × 0.4 cm) were taped on the plantar surface of both forelimbs, by applying equal pressure. Next, the mice were placed back in the test box. Two parameters were counted: time-to-contact, defined as the time required for the mouse to sense the presence of the adhesive (i.e., mouth to paw contact), indicative of correct paw and mouth sensitivity, and time-to-remove, defined as the time required to completely remove the adhesive from the paw, reflecting sensorimotor performance (Bouet et al., 2009). Furthermore, in order to obtain a more direct measure of skilled and coordinated forelimb use after adhesive contact, we subtracted time-to-contact from time-to-remove for each mouse (remove-contact), a factor that can also be influenced by motivational aspects. If the mouse did not feel or remove an adhesive during the trial, a maximum time of 120 s was given. Prior to surgery, the mice were trained for 5 days by performing the test in identical conditions as the test condition. The adhesive placement order (left forepaw or right forepaw first) was alternated for each day of training during the first 4 days, and randomized for the last day of training, and for the test session.

### Nest building test

Spontaneous motor performance, skilled forelimb use, and motivational aspects of behavior were investigated using the nest building test, as described previously (Deacon, 2006). In this paradigm, mice were individually housed overnight in a non-enriched cage, and challenged to build a nest starting from nesting material in order to provide shelter and heat insulation. The following morning, the quality of the nest was scored on a 0–5 scale, with 0 representing no nest, and 5 a perfect nest (Deacon, 2006). Furthermore, in order to provide a semi-independent objective assay of nesting ability, the amount of nesting material shredded was quantified, by weighing the complete nesting material (one pressed cotton Nestlet™ square) before the test, and weighing the untorn material at the end of the test.

### Open-field test

For the open-field test, each mouse was monitored for 60 min in a Plexiglas box (60 × 60 cm; height 60 cm; center of the arena defined as the central 40 × 40 cm zone), using an overhead video tracking system connected to Ethovision 3.0 (Noldus). At the end of a trial, various behavioral parameters were objectively integrated by the software, including motor parameters (distance traveled, velocity), as well as measures of perseverative motor behavior (turn angle, meander) and perseverative thigmotaxis/anxiety-like behavior (time spent in center). Additionally, the thigmotactic scanning behavior during the first 5 min of each trial was manually scored, by reviewing the recorded tracks in Ethovision, in a blinded manner. The thigmotactic scanning preference (ipsilateral or contralateral to the lesion) was analyzed by quantifying the time spent scanning the open-field walls either using the ipsilateral side, or contralateral side of the body.

### Light/dark test

In the light/dark test, anxious behavior is investigated by comparing the innate exploratory activity of the mice with the preference for an enclosed, safe shelter. Anxiety levels are revealed by two parameters: time spent outside the shelter, and the latency to exit the shelter. Additionally, using the set-up described by Pogorelov et al., motor behavior parameters can be simultaneously reported, by analyzing the velocity of the mouse outside the shelter (Pogorelov et al., 2007). At the beginning of the test, each mouse starts in a dark shelter (30 × 30 cm; height 8.5 cm) positioned in the corner of the open-field arena. Outside the shelter, a brightly lit environment was created using three overhead lamps, generating a light contrast (illuminance outside the shelter 700 lx, inside the shelter 0.5 lx). The behavior of the mouse during a 5 min trial was monitored using Ethovision. At the end of the test, the recorded tracks were manually integrated to reveal the time spent outside the shelter, as well as the latency to exit the shelter.

### Tail suspension test

Depressive-like behavior was investigated using the tail suspension test. In this “behavioral despair” paradigm, each mouse was suspended by the tail, and videotaped during 5 min. Integration of the tail suspension videos was performed by manually counting the total immobility time of each mouse. We also integrated on the same video recordings the side preference (ipsilateral vs. contralateral) when performing body swings during the escape-oriented behavior. This parameter, also reported in the elevated body swing test, is believed to give an indication of motor asymmetry in unilateral DA depletion models (Roghani et al., 2002). Body swings were quantified during the first minute of the tail suspension test. A swing was counted whenever the mouse displaced its body 30° from the vertical axis, and for another swing to be counted the mouse must have returned first to its vertical position. Mice that climbed their own tail during the test were excluded from the study.

### Neurochemical analysis of total DA and DOPAC content in the striatum

After behavioral analyses, mice were sacrificed by cervical dislocation. Brains were quickly removed, and the caudal part was post-fixed for 3 days in freshly prepared 4% paraformaldehyde for further use in immunohistochemistry (Sigma-Aldrich, Brussels, Belgium). From the rostral part of the brain, striata were dissected out on ice cold petri-dish, weighed, and homogenized in 400 μL antioxidant solution (0.05M HCl, 0.5% Na_2_S_2_O_5_, and 0.05% Na_2_EDTA) containing 10 ng/100 μl 3,4-dihydroxybenzylamine as internal standard. The homogenate was centrifuged for 20 min at 10 000 × g at 4°C, and the supernatants were immediately diluted 1:5 in 0.5 M acetic acid. Twenty μL of the resulting sample dilution were analyzed for DA and DOPAC content on a narrow-bore (C18 column, 5 μm, 150 × 2.1 mm; Altima Grace, Lokeren, Belgium) liquid chromatography system with an electrochemical detector (Antec, Leiden, The Netherlands), as described previously (Massie et al., 2011).

### Immunohistochemistry for detecting tyrosine hydroxylase (TH) and parvalbumin (PV) expressing neurons

Forty μm vibratome sections were cut from the post-fixed caudal part of the brain (Leica Microsystems, Germany), and stored in serial order in 10 mM PBS supplemented with 1.5 mM sodium azide at 4°C. Nigral sections were selected to investigate the presence of TH and PV expressing neurons, using rabbit anti-TH antibody (AB152; 1/2000; Millipore, Temecula, CA, USA) and rabbit anti-PV antibody respectively (PV 25; 1/10000; Swant, Bellinzona, Switzerland), and employing the ABC peroxidase technique, as described previously (Massie et al., 2011). Photomicrographs were taken of the stained sections, and cell counts were performed using ImageJ software (U.S. National Institutes of Health, Bethesda, MD, USA). Total numbers of TH+ profiles in the SNc and VTA were counted blindly in 6 serial sections throughout the entire rostro-caudal extent of the SN (every 3 sections, 1 section was stained). Regional sub-analysis of the VTA was performed by counting the number of TH+ profiles in the lateral VTA (parabrachial pigmented nucleus, PBP) in 3 serial sections covering the peri-injection site (AP −2.92 to −3.08 from bregma). Similarly, the total number of SNr PV+ profiles was evaluated in 3 serial sections covering the peri-injection site (AP -2.92 to -3.08 from bregma). The TH+ SN volume was determined by stereological volume measurements based on the Cavalieri method, on 4 serial TH stained sections covering the rostro-caudal extent of the SN (every 5 sections, 1 section was analyzed), using a computerized system (Stereo Investigator; MicroBright-Field, Germany).

### Immunodetection of α-synuclein and S129-P α-synuclein

Mice in which we investigated the effect of LAC administration on α-synuclein and S129-P α-synuclein were sacrificed by an overdose of pentobarbital (Nembutal, Ceva Sante Animale, Brussels Belgium), and transcardially perfused with NaCl 0.9% for 3 min, followed by freshly depolymerized 4% paraformaldehyde in NaCl 0.9% supplemented with 0.85% H_3_PO_4_ (pH 7.42) for an additional 6 min. Next, brains were removed and post-fixed in the same fixative overnight, rinsed in tap water for 24 h, and stored in 10 mM PBS supplemented with 1.5 mM sodium azide at 4°C. Free-floating 40 μm frontal sections were made with a vibratome and stored in serial order in 10 mM PBS supplemented with 1.5 mM sodium azide at 4°C. Sections were pre-treated with a mixture of 3% hydrogen peroxide and 10% methanol in TBS for 10 min followed by a blocking step of 1 h in 10% normal goat serum (DakoCytomation, Belgium) prepared in TBS. Next, sections were incubated overnight with primary antibody against α-synuclein (rabbit antibody, 1:5000, home-made, Anke Van der Perren and Veerle Baekelandt, unpublished) or S129-P α-synuclein (mouse monoclonal 1:5000, Elan Pharmaceuticals, Ireland) in TBS-0.1% triton X-100, 10% goat serum. As secondary antibody we used biotinylated anti-rabbit IgG for α-synuclein or biotinylated anti-mouse IgG for S129-P α-synuclein (both 1:600, DakoCytomation), followed by incubation with streptavidin–horseradish peroxidase complex (1:1000, DakoCytomation). Immunoreactivity was visualized using 3,3-diaminobenzidine (0.4 mg/ml, Sigma-Aldrich) as a chromogen. After being rinsed and mounted, sections were cover slipped using DPX mounting medium (Sigma-Aldrich). Photomicrographs were taken of the stained sections, and densitometric analyses were performed using ImageJ software in sections covering the entire rostro-caudal extent of the SN. Optical density values were calculated as [log265/mean gray value(SN)—log265/mean gray value(background)], where SN was defined as the combined SNc + SNr area, and normalization for background staining was done considering the optical density of the ipsilateral cerebral peduncle.

For fluorescent double staining, sections were rinsed three times in PBS and then incubated overnight in PBS-0.1% triton X-100, 10% donkey serum, and the following antibodies: mouse anti-S129-P α-synuclein (1:1000, Elan Pharmaceuticals), rabbit anti-TH (1:1000, Chemicon 152), rabbit anti-NeuN (1:1000, Millipore ABN78) and rabbit anti-GFAP (1:200, Dako, ZO344). After three rinses in PBS-0.1% triton X-100 the sections were incubated in the dark for 1 h in fluorochrome-conjugated secondary antibodies: donkey anti-mouse Alexa 488 (1:400, Molecular Probes™, Invitrogen, Belgium) and donkey anti-rabbit Alexa 647 (1:400, Molecular Probes™, Invitrogen). After being rinsed in PBS and mounted, the sections were coverslipped with mowiol. Fluorescent double staining was visualized by confocal microscopy with an LSM 510 unit (Zeiss, Belgium).

### Statistical analysis

Data are expressed as mean ± standard error of the mean (s.e.m.). Statistical analyses were performed using GraphPad Prism 4.0 software. For studying one variable within one group of animals (Table 1), we used Student's paired *t*-test. For all other analyses, we employed the Two-Way ANOVA followed by Bonferroni *post-hoc* test. The α-value was set at 0.05.

**Table 1:** **Neuropathological and neurochemical changes after 3 μg LAC (or saline) infusion in the left SNc**.

	**SHAM 1w**		**LAC 1w**		**SHAM 3w**		**LAC 3w**	
	**Ipsi**	**Contra**	**Ipsi**	**Contra**	**Ipsi**	**Contra**	**Ipsi**	**Contra**
TH+ profiles SNc	1071 ± 75.42	1175 ± 58.27	725 ± 29.74^***^	1298 ± 65.26	995.2 ± 108.3	1077 ± 87.75	742.8 ± 108^***^	1205 ± 69.67
TH+ profiles VTA	1630 ± 143.5	1655 ± 180.5	1431 ± 182.8	1696 ± 41.52	1491 ± 179.7	1656 ± 107.7	1562 ± 254.2	1618 ± 255.1
TH+ profiles PBP ^p.i.^	226.4 ± 14.8	250.8 ± 24.5	159.2 ± 12.1^*^	258.2 ± 29.5	208.7 ± 20.2	232.7 ± 15.8	163.3 ± 15.5^**^	245.7 ± 21.6
PV+ profiles SNr ^p.i.^	565.2 ± 34.9	625.4 ± 41.6	529.5 ± 26.7	569.8 ± 47.2	–	–	–	–
TH+ volume SN (mm^3^)	0.457 ± 0.015^*^	0.482 ± 0.017	0.367 ± 0.021^**^	0.452 ± 0.015	0.398 ± 0.024	0.406 ± 0.022	0.320 ± 0.014^***^	0.421 ± 0.017
α-synuclein SN ^OD^	1.04E-3 ± 1.9E-4	1.02E-3 ± 1.2E-4	2.53E-3 ± 3.6E-4^*^	1.56E-3 ± 2.5E-4	1.91E-3 ± 1.7E-4	1.75E-3 ± 2.2E-4	3.23E-3 ± 5.3E-4^*^	2.22E-3 ± 2.9E-4
α-synuclein S129-P SN ^OD^	2.96E-3 ± 2.4E-4	2.83E-3 ± 2.2E-4	4.39E-3 ± 8.3E-4^**^	1.83E-3 ± 5.9E-4	2.64E-3 ± 5.8E-4	2.48E-3 ± 4.9E-4	3.59E-3 ± 6.7E-4^**^	1.64E-3 ± 4.9E-4
DA (ng/g) striatum	13681 ± 1219	12875 ± 1014	9405 ± 1219^***^	15089 ± 1132	15460 ± 429	15746 ± 1435	9302 ± 1639^***^	15838 ± 1321
DOPAC (ng/g) striatum	1503 ± 252.0	1760 ± 399.3	957.4 ± 108.1^**^	1361 ± 119.2	1255 ± 65.66	1312 ± 90.80	1238 ± 309.5	1925 ± 436.1
DOPAC/DA striatum	0.125 ± 0.034	0.151 ± 0.044	0.107 ± 0.013	0.091 ± 0.007	0.081 ± 0.005	0.087 ± 0.009	0.169 ± 0.050	0.138 ± 0.042

Data are presented as mean ± s.e.m., and were analyzed using a paired t-test, comparing variables from left and right brain structures of the same mouse (

*** *p < 0.001*,

** *p < 0.01*,

* p < 0.05). DA, dopamine; DOPAC, 3,4-dihydroxyphenylacetic acid; LAC, lactacystin; PV, parvalbumin; SN, substantia nigra; SNc, substantia nigra pars compacta; SNr, substantia nigra pars reticulata; TH, tyrosine hydroxylase; VTA, ventral tegmental area (^p.i.^, peri-injection; ^OD^, optical density, calculated as described in Materials and Methods).

## Results

### Intranigral injection of LAC induces acute and non-progressive nigrostriatal DA-ergic degeneration

**Figure 1:**
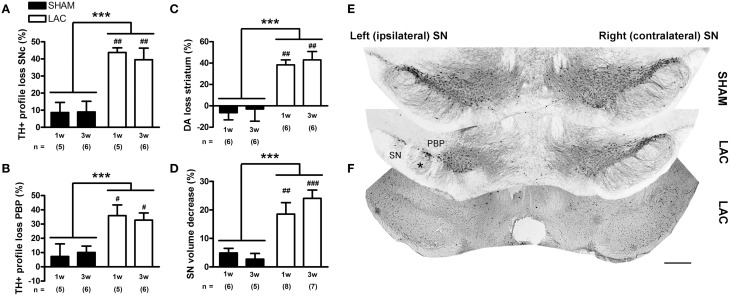
**Significant degeneration of the nigrostriatal DA-ergic pathway in mice receiving 3 μg LAC**. TH immunohistochemistry revealed a significant loss of TH+ profiles in the SNc **(A,E)**, as well as in the PBP located at the injection site **(B,E)**, following LAC administration. Loss of nigral TH+ profiles translated to reduced striatal DA content **(C)**. LAC lesion also caused a slight atrophy **(D)** and deafferentation of the ipsilateral SN (asterisk in **E**), but did not damage GABA-ergic neurons in the SNr at the injection site (as evaluated using PV immunohistochemistry, **F**). Data are presented as percentage decrease in TH+ profiles **(A,B)**, DA content **(C)**, or TH+ SN volume **(D)** compared to the intact side (mean ± s.e.m.). ***p < 0.001 (Two-Way ANOVA), ^###^*p* < 0.001, ^##^*p* < 0.01, ^#^*p* < 0.05 (Bonferroni *post-hoc* vs. sham). Sample size indicated in the figure. DA dopamine, LAC lactacystin, PBP parabrachial pigmented nucleus, SN substantia nigra, SNc substantia nigra pars compacta, TH tyrosine hydroxylase. Scale bar 400 μm **(E,F)**.

Immunohistochemical analyses revealed that 3 μg LAC administration led to significant loss of nigral TH expressing cells in the lesioned SNc compared to the contralateral intact SNc (Figures 1A,E, Table 1). Two-Way ANOVA (with time and treatment as factors) revealed a global effect of LAC lesion on loss of nigral TH+ profiles [treatment factor: *F*_(1, 18)_ = 31.12, *p* < 0.001], with a significant decrease of ~40% TH+ profiles in the ipsilateral SNc at both 1 week (*p* < 0.01) and 3 weeks (*p* < 0.01) post-surgery. No significant changes could be observed for TH+ profiles in the entire rostro-caudal extent of the VTA [treatment factor: *F*_(1, 18)_ = 0.21, *p* > 0.05; Table 1]. However, regional analyses of TH+ profiles in the lateral VTA (PBP) revealed a local loss of ~30% TH+ profiles [treatment factor: *F*_(1, 18)_ = 15.86, *p* < 0.001], at both 1 week (*p* < 0.05) and 3 weeks (*p* < 0.05) post-surgery, in sections covering the injection site (AP −2.92 to −3.08 from bregma) (Figures 1B,E, Table 1).

Loss of nigral TH+ profiles translated into a significant loss of DA content in the ipsilateral striatum compared to the contralateral striatum (Figure 1C, Table 1). Similar as for the nigral degeneration, Two-Way ANOVA revealed a global effect of LAC lesion on striatal DA content [treatment factor: *F*_(1, 20)_ = 32.54, *p* < 0.001], with a significant decrease of ~40% DA content in the ipsilateral striatum observed at both 1 week (*p* < 0.01) and 3 weeks (*p* < 0.01) post-surgery. No significant changes could be observed in striatal DOPAC/DA ratio at either 1 or 3 weeks after LAC (Table 1), indicating no changes in DA turnover.

Intranigral administration of LAC caused a small, but significant atrophy of the ipsilateral SN, as evaluated using the Cavalieri probe estimator (Figures 1D,E, Table 1). Two-Way ANOVA revealed a global effect of the lesion on the TH+ ipsilateral SN volume [treatment factor: *F*_(1, 22)_ = 30.00, *p* < 0.001], with a significant decrease of ~20% in volume observed at both 1 week (*p* < 0.01) and 3 weeks (*p* < 0.001) post-surgery.

Finally, in order to investigate possible non-specific effects of the lesion on GABA-ergic neurons located in the SNr, we compared the number of PV-expressing neurons in the ipsilateral SNr to the contralateral SNr on stained sections surrounding the injection site (covering AP −2.92 to −3.08 from bregma). We did not observe, however, any significant effects of the LAC lesion on PV+ neurons in the SNr in either the sham group or the LAC group, 1 week following surgery (Figure 1F, Table 1).

### Intranigral injection of LAC causes accumulation of α-synuclein and S129-P α-synuclein

**Figure 2:**
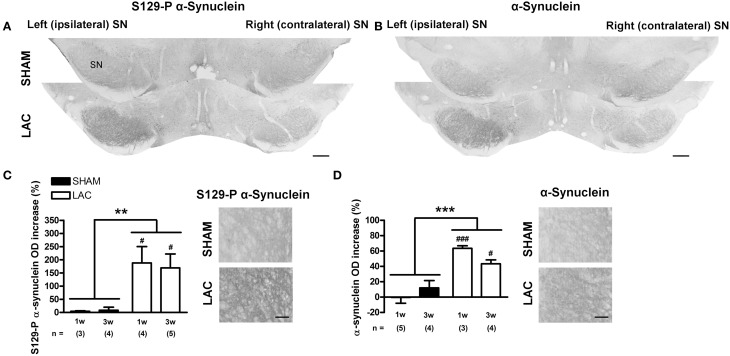
**Accumulation of α-synuclein and S129-P α-synuclein in the SN of mice receiving 3 μg LAC**. Immunohistochemical analyses revealed an increase in S129-P α-synuclein **(A,C)** and α-synuclein **(B,D)** immunoreactivity in the SN of LAC-treated mice. High magnification photomicrographs of the ipsilateral SNr in **(A,B)** demonstrate the presence of S129-P α-synuclein and α-synuclein immunoreactive fibers (**C,D** respectively). Data are presented as percentage increase in S129-P α-synuclein **(C)** or α-synuclein **(D)** optical density compared to the intact side (mean ± s.e.m.). ***p < 0.001, **p < 0.01 (Two-Way ANOVA), ^###^*p* < 0.001, ^#^*p* < 0.05 (Bonferroni *post-hoc* vs. sham). Sample size indicated in the figure. LAC lactacystin, OD optical density, S129-P S129-phosphorylated, SN substantia nigra. Scale bar 400 μm **(A,B)**, 50 μm **(C,D)**.

**Figure 3:**
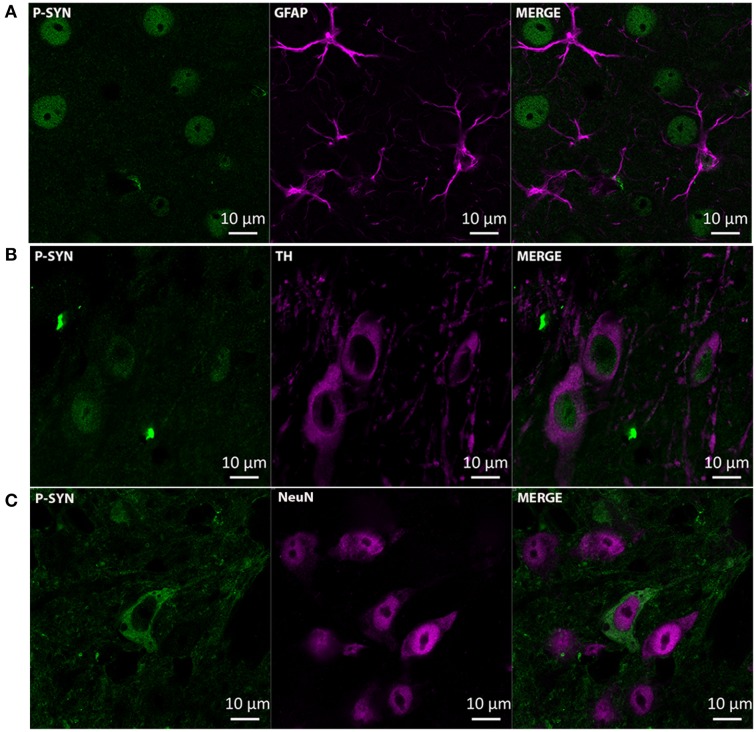
**Accumulation of S129-P α-synuclein in SNc neurons of mice receiving 3 μg LAC**. Immunofluorescence analyses demonstrate accumulation of S129-P α-synuclein in nigral TH+ **(B)** and NeuN+ **(C)** neurons, but not GFAP+ astrocytes **(A)**. GFAP, glial fibrillary acidic protein; NeuN, neuronal nuclei; TH, tyrosine hydroxylase. Scale bar 10 μm.

Immunohistochemistry revealed that intranigral administration of LAC led to an increase in α-synuclein (Figures 2B,D) and S129-P α-synuclein (Figures 2A,C) immunoreactivity at both 1 and 3 weeks post-lesion, with no signs of progression of pathology between the two time points. Densitometric analyses revealed an increase in α-synuclein immunoreactivity in the ipsilateral SN with ~50% [treatment factor: *F*_(1, 12)_ = 40.20, *p* < 0.001], that could be observed at both 1 week (*p* < 0.001) and 3 weeks (*p* < 0.05) post-lesion (Figure 2D, Table 1). Furthermore, we could document an increase in S129-P α-synuclein immunoreactivity in the ipsilateral SN with ~150% [treatment factor: *F*_(1, 12)_ = 13.23, *p* < 0.01], at both 1 week (*p* < 0.05) and 3 weeks (*p* < 0.05) post-surgery (Figure 2C, Table 1). The increased expression of α-synuclein and S129-P α-synuclein could be observed throughout the entire rostro-caudal extent of the SN, and did not affect neighboring regions, such as the VTA (Supplementary Figure 1). Furthermore, the increased immunoreactivity seemed to be observed mainly in the SNr, where it presented as neuropil (Figures 2C,D). Confocal fluorescent double labeling experiments also indicated increased S129-P α-synuclein expression in SNc neurons, as S129-P α-synuclein was seen to accumulate in TH+ (Figure 3B) and NeuN+ (Figure 3C) nigral neurons, but not GFAP+ astrocytes (Figure 3A). Accumulated S129-P α-synuclein showed both nuclear (Figure 3B), and cytoplasmic (Figure 3C) subcellular distribution.

### LAC-treated mice develop parkinsonian motor and non-motor features

For all behavioral tests described, Two-Way ANOVA failed to reveal any interaction effect between time and lesion, indicating a stable and non-progressive behavioral phenotype between 1 and 3 weeks. Furthermore, all behavioral tests were conducted in a drug-free state, which is believed to give a natural and relevant behavioral outcome in models of nigrostriatal degeneration (Cenci et al., 2002; Glajch et al., 2012).

**Figure 4:**
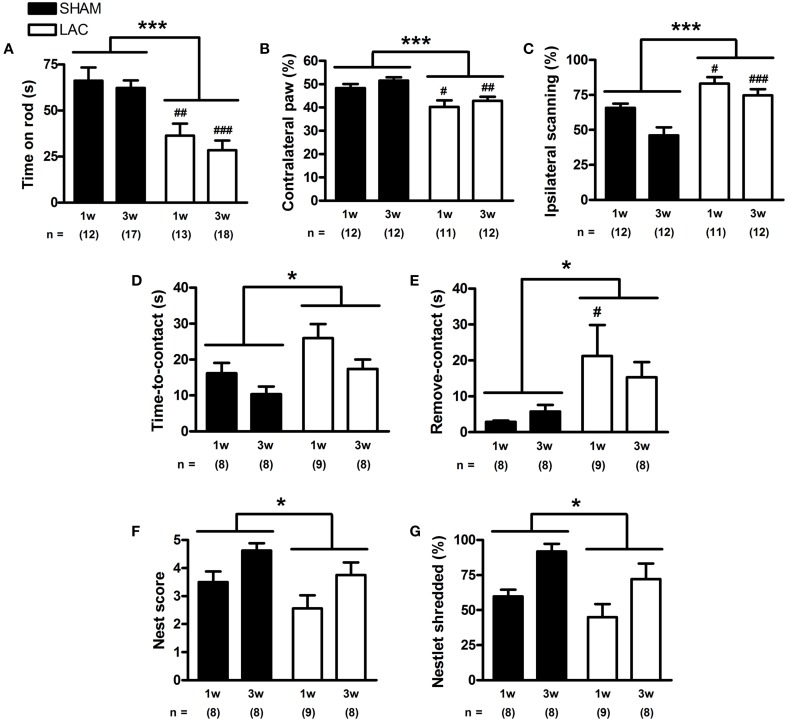
**Motor and somatosensory impairment in mice receiving 3 μg LAC**. In the accelerating rotarod test, LAC lesioned mice showed decreased time spent on the rod, compared to sham mice receiving vehicle **(A)**. In the cylinder test, LAC-treated mice engaged in significantly less right paw wall-contacts compared to sham mice **(B)**. Also, LAC-treated mice had an increased preference to scan the cylinder walls more with their intact ipsilateral side **(C)**. In the adhesive removal test, both time-to-contact (**D**), and the time required to remove the adhesive after detection (time-to-remove—time-to-contact) **(E)**, were globally increased after LAC treatment compared to sham mice. Furthermore, in the nest building test, the nest score **(F)**, as well as the amount of nesting material shredded **(G)**, were globally decreased after LAC lesion, compared to sham mice. Data are presented as mean ± s.e.m. *p < 0.05, ***p < 0.001 (TwO-Way ANOVA), ^#^*p* < 0.05, ^##^*p* < 0.01, ^###^*p* < 0.001 (Bonferroni *post-hoc* vs. sham). Sample size indicated in the figure. LAC lactacystin.

First, we investigated the effect of the LAC lesion on motor function, using the accelerating rotarod task, a classical test used to assess motor coordination and balance in rodents bearing DA lesions. This paradigm is also believed to give indications of bradykinesia and/or limb rigidity, as the accelerating protocol requires fast and continuous adaptation of the mouse to increasing rod speeds (Sedelis et al., 2001). No significant differences could be observed in baseline performance in the training phase between groups (Supplementary Figure 2). After surgery, LAC-treated mice demonstrated decreased rotarod performance when compared to sham mice [treatment factor: *F*_(1, 56)_ = 31.15, *p* < 0.001], an effect that could be significantly observed at both 1 week (*p* < 0.01) and 3 weeks (*p* < 0.001) post-lesion (Figure 4A).

Next, we employed the cylinder test to investigate forelimb asymmetry, and revealed that LAC-treated mice had a significant decrease in the use of the contralateral paw for performing weight-bearing supporting touches while exploring the cylinder walls [treatment factor: *F*_(1, 42)_ = 18.70, *p* < 0.001]. This small, but significant effect could be observed at both 1 week (*p* < 0.05) and 3 weeks (*p* < 0.01) after lesion, indicating contralateral forelimb akinesia (Figure 4B). Moreover, blinded investigation of cylinder test video recordings revealed that LAC-treated mice showed an increased preference to scan the cylinder walls using the ipsilateral side of the body [treatment factor: *F*_(1, 45)_ = 25.26, *p* < 0.001] (Figure 4C), that was seen to significantly occur at both 1 week (*p* < 0.05) and 3 weeks (*p* < 0.001) post-lesion.

Using the adhesive removal test, we investigated the effect of LAC lesion on sensorimotor function. Baseline performance demonstrated no significant differences in the training phase between groups (Supplementary Figure 2). After lesion, we could notice a global increase in time-to-contact [treatment factor: *F*_(1, 29)_ = 7.58, *p* < 0.05] (Figure 4D), indicating a slightly decreased sensitivity to tactile stimuli. The adhesive removal results also demonstrated a global impairment of skilled and coordinated forelimb use [treatment factor: remove-contact, *F*_(1, 29)_ = 7.12, *p* < 0.05], that seemed to be particularly visible at 1 week post-lesion (*p* < 0.05). This impairment affected the contralateral paw (Figure 4E), with no significant differences observed in the performance of the ipsilateral paw (data not shown), indicating a specific effect of the unilateral LAC lesion on skilled forelimb use and/or motivation to remove the adhesive.

To gain more insight into the effect of LAC on skilled forelimb performance, we employed the nest building test. After LAC lesion, we could observe a slight and global decrease in the nest score when compared to sham-treated mice [treatment factor: *F*_(1, 29)_ = 4.98, *p* < 0.05] (Figure 4F). This effect was also observed when weighing the amount of nesting material shredded overnight, as a semi-independent objective assay of nesting ability [treatment factor: *F*_(1, 29)_ = 4.36, *p* < 0.05] (Figure 4G).

**Figure 5:**
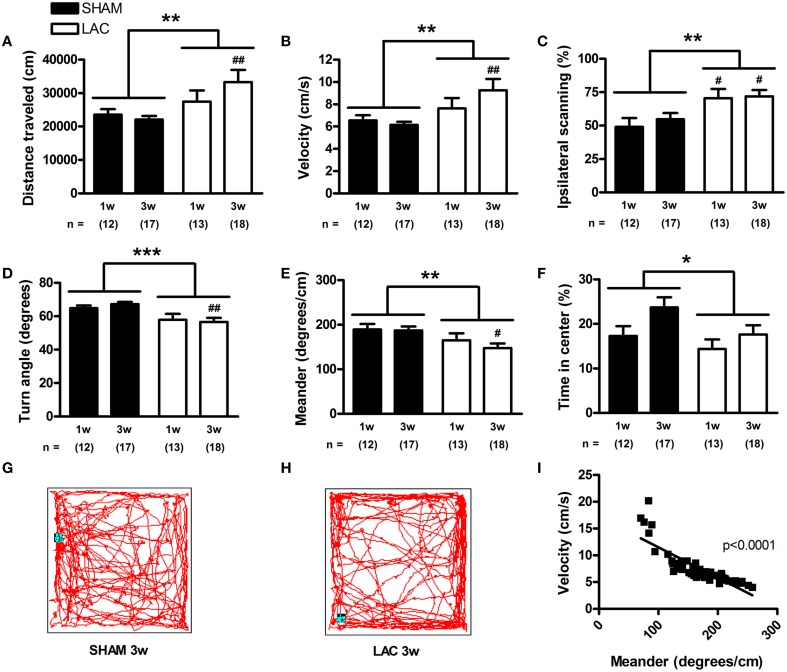
**Spontaneous behavior of mice treated with 3 μg LAC as observed in a 60 min open-field test**. Distance traveled **(A)**, and velocity **(B)** were globally increased in LAC lesioned mice, compared to sham mice receiving vehicle. Furthermore, mice treated with LAC had significantly increased ipsilateral scanning behavior **(C)**. LAC lesion also induced global decreases in turn angle **(D)**, as well as in meander **(E)**. This perseverative behavior is reflected in representative 10 min trajectories of a sham-treated **(G)**, and a LAC-treated **(H)** mouse at 3 weeks post-surgery. Linear regression analysis performed with data from all experimental groups revealed a close inverse correlation between meander (perseverative behavior) and velocity (hyperkinesia) **(I)**. LAC lesion induced a global reduction in time spent in the center of the arena, compared to sham mice **(F)**. Data are presented as mean ± s.e.m. *p < 0.05, **p < 0.01, ***p < 0.001 (Two-Way ANOVA), ^#^*p* < 0.05, ^##^*p* < 0.01 (Bonferroni *post-hoc* vs. sham). Sample size indicated in the figure. LAC lactacystin.

In order to evaluate spontaneous activity, we employed the open-field test. Surprisingly, the results revealed a general hyperkinetic behavior when integrating both distance traveled [treatment factor: *F*_(1, 56)_ = 7.22, *p* < 0.01] (Figure 5A) and velocity [treatment factor: *F*_(1, 56)_ = 7.21, *p* < 0.01] (Figure 5B), that was especially visible 3 weeks post-surgery (*p* < 0.01). The observed hyperactive behavior could be due to the development of akathisia after lesion, a feature that also occurs in early PD patients (Gjerstad et al., 2011). Indeed, we noticed increased restlessness of LAC-treated mice during the tail suspension test. While sham-treated mice exhibited a normal behavior during the tail suspension test, with periods of immobility after the initial struggle to escape (Supplementary Video 1, showing a representative 1 min interval of the tail suspension test starting at 2 min and 30 s into the test), LAC-treated mice demonstrated continuous movement/restlessness during the same period of time (Supplementary Video 2 for minor akathisia-like behavior, Supplementary Video 3 for strong akathisia-like behavior). As such, development of akathisia could have masked potential bradykinetic effects of LAC on behavior in the open-field test. Also in the open-field test we could observe an increased ipsilateral thigmotactic behavior after blinded integration of the first 5 min of the trial. LAC lesion led to an increased preference to scan the open-field walls using the ipsilateral side of the body [treatment factor: *F*_(1, 56)_ = 11.46, *p* < 0.01] (Figure 5C), at both 1 week (*p* < 0.05) and 3 weeks (*p* < 0.05) post-lesion. Finally, analysis of open-field data revealed that LAC-treated mice demonstrate global and slight decreases in turn angle [treatment factor: *F*_(1, 56)_ = 13.58, *p* < 0.001] (Figure 5D), as well as in meander [treatment factor: *F*_(1, 56)_ = 7.32, *p* < 0.01] (Figure 5E), that were particularly evident 3 weeks post-lesion (*p* < 0.01 for turn angle, and *p* < 0.05 for meander). These results indicate that lesioned mice tended to follow more straight, repetitive motor sequences, with decreased turning behavior (Figures 5G,H). Interestingly, performing linear regression analysis with data from all experimental groups showed that meander was inversely correlated with velocity in the open-field, highlighting that perseverative behavior and hyperkinesia are closely related and potentially inter-connected as behavioral phenotypes following LAC (Figure 5I).

**Figure 6:**
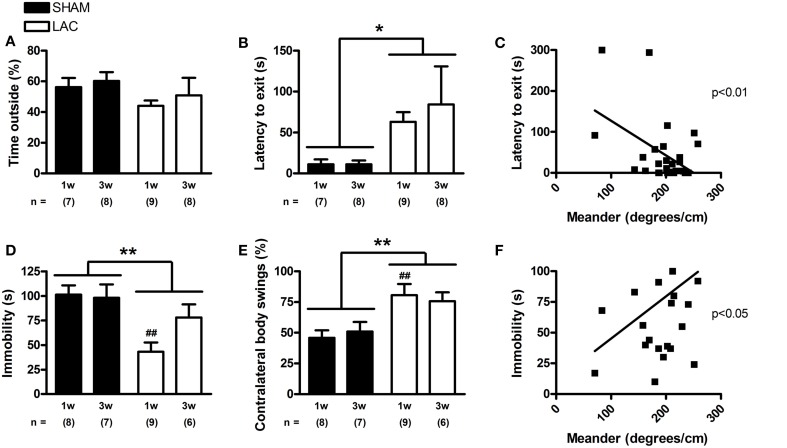
**The light/dark and tail suspension tests in mice receiving 3 μg LAC**. Although the time spent outside the shelter in the light/dark paradigm was not significantly affected **(A)**, LAC lesion led to a global increase in the latency to exit the shelter compared to sham mice receiving vehicle **(B)**. The tail suspension test revealed that LAC-treated mice had decreased periods of immobility, compared to sham mice **(D)**. Linear regression analysis performed with data from all experimental groups revealed that perseverative behavior (meander) correlates with latency to exit the shelter **(C)**, as well as with the immobility time in the tail suspension tests **(F)**. Mice treated with LAC also demonstrated significantly increased ipsilateral body swings compared to sham mice during their escape-oriented behavior **(E)**. Data are presented as mean ± s.e.m. *p < 0.05, **p < 0.01 (Two-Way ANOVA), ^##^*p* < 0.01 (Bonferroni *post-hoc* vs. sham). Sample size indicated in the figure. LAC lactacystin.

Depression and anxiety are highly relevant parkinsonian non-motor symptoms, and important determinants for the quality of life in PD (Martinez-Martin, 2011). Analysis of the time spent in the center of the open-field arena revealed that LAC lesion induced a slight and global decrease in the time spent in center [treatment factor: *F*_(1, 56)_ = 4.08, *p* < 0.05] (Figure 5F). In the light/dark test, LAC lesion led to a global increase in latency to exit the shelter [treatment factor: *F*_(1, 28)_ = 6.44, *p* < 0.05] (Figure 6B), but did not influence the time spent outside the shelter [treatment factor: *F*_(1, 28)_ = 2.25, *p* > 0.05] (Figure 6A). These changes occurred in the absence of any effect on motor behavior, as open-field video tracking of the mouse while outside the shelter failed to reveal any significant changes in velocity (data not shown). The significant effect of LAC lesion on the latency to exit the shelter, but not the time spent outside the shelter was intriguing, and we hypothesized that the observed effects could also reflect perseverative thigmotaxis. Indeed, we could notice a significant inverse correlation between latency to exit the shelter and meander in the open-field (Figure 6C), indicating that the increased latency to exit the shelter can be due to perseverative behavior causing decreased alternations between the two compartments of the open-field. Perseverative thigmotaxis could have also manifested in the open-field test, leading to a global decrease in time spent in center.

Finally, we investigated the effect of LAC administration on development of depressive-like symptoms using the mouse tail suspension test. Interestingly, in sharp contrast to previous findings in the literature (Drui et al., 2014), we could observe a global, paradoxical decrease in immobility time after LAC [treatment factor: *F*_(1, 26)_ = 11.83, *p* < 0.01] (Figure 6D) that seemed to be especially evident at 1 week post-lesion (*p* < 0.01). However, several factors might have biased the interpretation of this test. First, the development of a hyperkinetic behavior and restlessness after LAC lesion could have confounded the effect on immobility time. Furthermore, the increased perseverative behavior documented after LAC could also influence the interpretation of the results, as the mice might have shown increased escape-oriented behavior due to cognitive vs. depressive-like behavioral differences. This is consistent with a significant correlation that we could find between immobility time in the tail suspension test and meander in the open-field test, highlighting that increased perseverative behavior can decrease immobility time in the tail suspension test (Figure 6F).

We have also used the tail suspension test as a platform to study motor asymmetry upon engaging in escape-oriented behaviors. Blinded analysis of the first minute from the tail suspension video tracks revealed that LAC lesion led to a global increase in contralateral body swings [treatment factor: *F*_(1, 26)_ = 13.61, *p* < 0.01] (Figure 6E), an effect especially visible at 1 week post-surgery (*p* < 0.01), confirming motor asymmetry following lesion.

## Discussion

Despite important advances in our understanding of the molecular pathogenesis and symptomatology of PD, there is still an urgent need for new developments, especially concerning translation of successful neuroprotective findings from pre-clinical animal studies to the clinic (Kieburtz and Olanow, 2007). Failure to achieve clinical translation might stem from various reasons, but it has been hypothesized that one could be the limited diversity of animal models used (Jenner, 2008; Duty and Jenner, 2011; Dexter and Jenner, 2013). Indeed, the molecular pathways relevant for PD pathogenesis are numerous and highly inter-connected, and include oxidative stress, mitochondrial dysfunction, protein aggregation, failure of protein degradation systems, neuroinflammation, and glutamate excitotoxicity (Dexter and Jenner, 2013; Lim and Zhang, 2013). Therefore, animal models mimicking any of these pathogenic pathways leading to DA-ergic neurodegeneration are highly relevant in terms of construct validity, and should probably be used in a combinatorial manner in pre-clinical drug development (Dawson et al., 2002). Furthermore, although α-synuclein pathology is a central feature in PD, it has not been consistently reproduced in classic toxin-based models of PD, such as the 6-hydroxydopamine (6-OHDA) or MPTP models (Dexter and Jenner, 2013). Although viral vector-based models overexpressing α-synuclein in the SN of rats or mice have been developed that display α-synuclein aggregation and dopaminergic cell death (Lauwers et al., 2007; Oliveras-Salva et al., 2013; Van der Perren et al., 2015), there is still an important need for the characterization of novel models of sporadic PD, with different mechanisms of action and good construct, face, and predictive validity (Duty and Jenner, 2011).

Recently, a contribution of proteasomal impairment to the protein aggregation and neurodegeneration associated with PD has been proposed (Olanow and McNaught, 2006; Lim, 2007; Ebrahimi-Fakhari et al., 2012). Post-mortem data obtained from sporadic PD indicated structural and functional defects in the 26/20S proteasome, including loss of 20S core α-subunits (McNaught et al., 2002a; Grunblatt et al., 2004; Bukhatwa et al., 2010), loss of 19S regulatory particles, including the proteasomal activator PA700 (McNaught et al., 2003; Grunblatt et al., 2004), as well as a general loss of all three peptidase activities of the 20S proteasome (McNaught and Jenner, 2001; Tofaris et al., 2003). In the brain, the proteasome seems to be specifically inhibited in the SNc, with sparing of proteasome function in other areas such as the striatum, or cortex, where neuronal loss is not observed (Furukawa et al., 2002; McNaught et al., 2003; Tofaris et al., 2003). Testing the proteasomal inhibition hypothesis of neurodegeneration *in vivo* requires the use of a highly specific and potent inhibitor that can inhibit all three peptidase activities of the proteasome, but does not affect other proteases or degradation pathways of the cell. This can be achieved using LAC, a Streptomyces natural product (Fenteany and Schreiber, 1998). Local administration of LAC to the nigrostriatal pathway has been previously shown to cause DA-ergic neurodegeneration when applied to the SNc of rats (McNaught et al., 2002b; Vernon et al., 2010; Mackey et al., 2013; Pienaar et al., 2015) or the medial forebrain bundle of mice (Xie et al., 2010).

In order to further describe the proteasome inhibition model of PD, we stereotaxically injected 3 μg LAC to the left SNc of mice. Our results demonstrated an acute loss of TH+ profiles in the injected SNc compared to the contralateral SNc of ~40%, that did not progress between 1 and 3 weeks post-surgery. In comparison to nigral neurons, neighboring VTA DA-ergic neurons did not seem to be significantly affected, when counted over the entire rostro-caudal extent. This particular resistance of VTA DA-ergic neurons to LAC-induced toxicity is in line with previous observations in LAC models (Xie et al., 2010; Mackey et al., 2013), and could be linked to the increased resilience of these DA-ergic neurons to toxic stimuli (Brichta and Greengard, 2014). Indeed, in a recent study, Subramaniam et al. demonstrated that direct infusion of proteasome inhibitor epoxomicin in the VTA of mice did not cause loss of VTA TH+ neurons, further hinting at a resistance of these neurons to proteasomal-induced cell death (Subramaniam et al., 2014). At the same time, however, Vernon et al. reported that after intranigral LAC administration, VTA DA-ergic neurons are not free of toxicity especially around the injection tract (Vernon et al., 2010). Therefore, we specifically re-evaluated the lateral part of the VTA, the PBP, at the level of the injection site. We chose to focus on this region for two important reasons: (i) it is located the closest to the injection coordinates, and (ii) it is the region of the VTA which is particularly affected in PD (McRitchie et al., 1997), indicating a potential vulnerability of this A10 cell subgroup to neurodegeneration. We found that LAC administration did damage this subset of neurons, leading to a loss of about 30% compared to the contralateral side, at both 1 and 3 weeks post-lesion. This effect was however not observed anymore when counting the entire rostro-caudal VTA, indicating a local effect of the lesion on the anterior PBP at the injection site.

Loss of nigral DA-ergic neurons translated to a significant loss of ~40% striatal DA, that did not progress between 1 and 3 weeks post-surgery. We did not detect any significant changes in DOPAC/DA ratio in the ipsilateral striatum after LAC. Increased DA turnover in the caudate/putamen is believed to be one of the functional compensatory changes occurring in PD (Pifl and Hornykiewicz, 2006), that maintains normal motor function until a threshold of ~80% DA depletion is reached. The observation that no changes in DA turnover occurred after intranigral LAC indicates the presence of an acute neurodegenerative process that occurred in the absence of functional compensation. The acute and non-progressive nature of the lesion is in line with previous reports of intranigral toxin models, such as the intranigral 6-OHDA rat model (Jeon et al., 1995) and the intranigral LAC rat model (Mackey et al., 2013), and might be related to a direct effect of the toxin on nigral DA-ergic cell bodies.

Stereological analysis of the TH+ ipsilateral SN volume revealed a significant shrinkage in volume of the SN after LAC lesion. A similar loss of planimetric (regional) volume of the TH+ ipsilateral SN, ~20% vs. the contralateral side, has been recently reported after moderate unilateral DA depletion induced by intrastriatal 6-OHDA in rats (Healy-Stoffel et al., 2014). Furthermore, our findings are in line with MRI findings documenting a decrease in the volume of the ipsilateral midbrain after administration of LAC to the SNc (Vernon et al., 2010) or the medial forebrain bundle (Vernon et al., 2011), as well as with the observation of decreased SN volume in PD patients (Sako et al., 2014). As the decrease in volume in our case did not seem to occur with loss of GABA-ergic neurons in the SNr, it is tempting to hypothesize that it might have resulted from secondary morphological changes, such as the deafferentation of the ipsilateral SNr due to loss of nigral DA-ergic dendrites.

Mechanistic studies demonstrated that LAC leads to DA-ergic cell death via apoptosis mediated by various upstream factors including reactive oxygen species (Perez-Alvarez et al., 2009), disturbance of iron (Vernon et al., 2010; Li et al., 2012; Le, 2014) and calcium (Li et al., 2007) homeostasis, and accumulation of p53 (Nair et al., 2006). LAC might also induce DA-ergic cell death, at least partly, by increasing the expression of S129-P α-synuclein, a post-translational modification present in ~90% of α-synuclein in Lewy bodies (Fujiwara et al., 2002; Anderson et al., 2006). Although the precise contribution of S129-P α-synuclein to the pathogenesis of PD remains to be elucidated (Tenreiro et al., 2014), this modification has recently been shown to enhance DA-ergic neurodegeneration in a rat model of familial PD (Sato et al., 2011), providing clues to a potential toxic gain-of-function. This is in line with the observation that S129-P α-synuclein expression increases progressively and concomitantly with the neurodegenerative degree in mice overexpressing α-synuclein (Oliveras-Salva et al., 2013). While accumulation of proteins such as S129-P α-synuclein, might be expected to cause toxicity, sub-lethal doses of proteasomal inhibitors given in combination with MPP+, rotenone, or 6-OHDA enhance the formation of α-synuclein positive inclusions, and surprisingly protect against neuronal death induced by either of the three toxins (Sawada et al., 2004; Inden et al., 2005). These findings support a role for low-level proteasomal inhibition in neuroprotection, potentially by allowing the formation of larger α-synuclein aggregates, which might be less toxic compared to smaller oligomeric species (Tanaka et al., 2004; Inden et al., 2005). In contrast, higher and toxic doses of proteasomal inhibitors exacerbate cell death induced by MPP+, rotenone, or 6-OHDA (Hoglinger et al., 2003), suggesting that the level of inhibition of the proteasome activity might dictate neuroprotection vs. enhanced cell death following toxic stimuli. In the current study, we observed that intranigral proteasomal inhibition in mice leads to increased S129-P α-synuclein immunoreactivity in the ipsilateral SN. This was accompanied by a similar increase in total levels of unmodified α-synuclein, consistent with previous findings indicating that unmodified α-synuclein is a substrate of the proteasome (Tofaris et al., 2001; Ebrahimi-Fakhari et al., 2011). Taking into account the aforementioned observations, it is difficult to resolve in our set-up whether α-synuclein and S129-P α-synuclein accumulation plays a toxic or protective role in LAC-induced DA-ergic cell death, or is simply an epiphenomenon following the lesion. Future investigations in this direction would be particularly informative in revealing the complex relationship between proteasomal inhibition, accumulation of α-synuclein, and DA-ergic cell death. Moreover, due to the general increase in α-synuclein, we cannot exclude that the increased expression of S129-P α-synuclein was simply due to accumulation of substrate available for phosphorylation. At the same time, however, Machiya et al. previously reported a specific increase in S129-P α-synuclein after proteasomal inhibition in the absence of changes in the total amount of α-synuclein, suggesting that S129-P α-synuclein is on its own a substrate for proteasomal degradation (Machiya et al., 2010). As such, the increased expression of S129-P α-synuclein in our case might be the combined result of impaired degradation of the phosphorylated protein and accumulation of substrate available for phosphorylation.

Increased immunoreactivity of α-synuclein and S129-P α-synuclein in immunohistochemistry was observed mainly in the SNr, where it presented as neuropil. Interestingly, Miwa et al. reported a similar distribution of increased expression of α-synuclein in nerve terminals in the SNr following intrastriatal administration of LAC to rats (Miwa et al., 2005). The authors suggested that this increase could be due to accumulation of α-synuclein in striatonigral terminals in the SNr (Miwa et al., 2005). In accordance, the observed effect at the level of the SNr after intranigral LAC might be due to an increase in α-synuclein and S129-P α-synuclein expression in nerve terminals projecting to the SNr. Furthermore, immunofluorescence analysis of the SNc revealed accumulation of S129-P α-synuclein in nigral neurons. Increased expression of S129-P α-synuclein was detected in TH+ and NeuN+ neurons, but not in GFAP+ astrocytes, highlighting a preferential effect of LAC in disrupting α-synuclein homeostasis in neurons. This is consistent with recent findings indicating that neurons have a lower proteasome activity compared to glial cells, and therefore are more vulnerable to protein accumulation (Tydlacka et al., 2008). It is noteworthy that we could also detect accumulation of S129-P α-synuclein in TH- neurons (data not shown). This is most likely the result of the indiscriminate mechanism of cell entry of the active metabolite of LAC (clasto-lactacystin β-lactone) that gains access to the 20S proteasome via passive diffusion through the cell membrane (Dick et al., 1997). In a similar manner, LAC was found to lead to formation of ubiquitin- and α-synuclein-positive inclusions in both TH+ and TH- neurons in rat ventral midbrain cultures (Rideout et al., 2005). Interestingly, accumulated S129-P α-synuclein had both cytoplasmic and nuclear distribution. The subcellular localization of α-synuclein is known to be mediated by S129 phosphorylation, with various kinases modulating the bi-directional shuttling of α-synuclein between nuclear and cytoplasmic compartments (Goncalves and Outeiro, 2013). Our findings of nuclear localization of S129-P α-synuclein are consistent with the observed nuclear distribution of S129-P α-synuclein in the SN of α-synuclein transgenic mice (Wakamatsu et al., 2007).

Several behavioral paradigms have been used to characterize motor and non-motor impairment in our model. Overall, mice injected intranigrally with LAC demonstrated PD-like motor behavioral impairment, consistent with previous studies investigating the effect of LAC lesions in rats (Vernon et al., 2010, 2011; Mackey et al., 2013; Konieczny et al., 2014; Pienaar et al., 2015), and mice (Xie et al., 2010). Both rotarod and cylinder tests revealed a significant decrease in motor performance in LAC-treated mice, reflecting impaired coordination and balance, bradykinesia, and contralateral forelimb akinesia. In the adhesive removal test, LAC lesion induced a general delay in the removal of the adhesive after contact, an effect that was especially visible at 1 week post-surgery. This impairment could reflect impaired skilled and coordinated movement, however it could also result from decreased motivation to remove the adhesive. The nest building test indicated a modest and global effect on the quality of the nest, as well as on the amount of nesting material shredded, demonstrating a slightly decreased capacity of the LAC mice to create a nest. This in turn, could be a consequence of decreased skilled forelimb use, however, similar as for the adhesive removal test, motivational aspects cannot be completely dissociated.

Besides motor symptoms, non-motor symptoms are increasingly recognized to be present and to have a very important impact on the quality of life of PD patients (Chaudhuri and Schapira, 2009). As non-motor symptoms are increasingly recognized in PD, there is a current need for their characterization in animal models (Dunnett and Lelos, 2010; Taylor et al., 2010). Although proteasome inhibition models have been shown to develop motor impairment, no studies were performed so far studying non-motor symptoms.

In order to address this issue, we first assessed the sensitive and somatosensory functions, as they are also affected in the human condition (Conte et al., 2013; Patel et al., 2014). In the adhesive removal test, we could observe a slightly decreased tactile sensitivity following LAC lesion, potentially indicating altered tactile sensory processing. Furthermore, evaluation of thigmotactic scanning behavior in the open-field and cylinder tests indicated that LAC lesion led to an increased preference to scan different enclosure walls using the ipsilateral intact side of the body. Increased ipsilateral thigmotactic scanning behavior has been documented in models of unilateral DA depletion (Steiner et al., 1988; Fornaguera et al., 1993), and is believed to reflect decreased responsiveness to sensory stimuli on the side of the body contralateral to the lesion (Huston et al., 1990). Alternatively, the increased ipsilateral scanning behavior might have resulted from spontaneous contralateral turning asymmetry after lesion. Interestingly, it was recently demonstrated that intranigral epoxomicin administration in mice leads to contralateral turning asymmetry, potentially linked with a hyperactive phenotype of the surviving DA neurons (Subramaniam et al., 2014). While it is difficult to resolve in the current set-up whether the increased thigmotactic scanning is purely linked with somatosensory disturbances (such as decreased somatosensory perception in the vibrissae contralateral to the lesion, Huston et al., 1990), the observation that LAC-treated mice show contralateral spontaneous turning in the center of the open-field arena, thereby without direct contact to the arena walls (unpublished observations), supports the view that it might arise at least partially as a result of an hyperactive phenotype of the surviving DA neurons. Furthermore, intranigral LAC injection resulted in a global hyperkinetic behavior, as observed in the open-field test, an effect especially visible at 3 weeks post-surgery. The surprising hyperactive behavior, also observed in the MPTP mouse model (Wang et al., 2013), might indicate akathisia, a sensory disturbance inducing a general restlessness, with a continuous urge to move, but of unknown pathophysiology (Patel et al., 2014). While akathisia seems to be present in PD (Lang and Johnson, 1987; Comella and Goetz, 1994; Gjerstad et al., 2011) and linked with disturbed DA-ergic transmission (Patel et al., 2014), this sensory disturbance, as well as the mechanisms associated, have not been fully explored in animal models. Interestingly, we could observe that LAC-treated mice exhibited increased restlessness during a period of the tail suspension test in which control mice demonstrate immobility, implying a possible development of akathisia-like behavior following LAC.

Impairments in cognitive flexibility are recognized as part of the executive dysfunction symptoms associated with early PD (Cools et al., 2001; Pagonabarraga and Kulisevsky, 2012). Deficits in set shifting (Cools et al., 2001), as well as an increased perseverative behavior (Ebersbach et al., 1994) have been reported to occur in early phases of PD. These cognitive impairments could have a strong impact on the quality of life as deficits in set shifting are strongly associated with freezing of gait (Naismith et al., 2010). Interestingly, LAC-treated mice demonstrated a global increase in perseverative behavior in the open-field, an effect especially visible 3 weeks post-surgery. The decreases in turn angle and meander indicated that lesioned mice generated less random movement, and tended to follow more straight sequences. These results are consistent with the modulating role played by striatal DA in controlling cognitive flexibility (Costa et al., 2014; Darvas et al., 2014).

Furthermore, the perseverative behavior was strongly manifested in our model, as it influenced the evaluation of mice in the anxiety- and depression-based paradigms. Indeed, in the light/dark test lesioned mice had an increase in the latency to exit the shelter that occurred in absence of changes in time spent outside the shelter. Together with a significant correlation with meander as measure of perseverative behavior, the increased latency to exit the shelter can be partly attributed to a decrease in alternations between the two compartments of the open-field due to perseverative thigmotaxis. Concomitantly, in the tail suspension test we could notice that LAC lesion led to a surprising global decrease in immobility time, an effect especially visible at 1 week post-surgery. These results are in sharp contrast to literature findings that indicate a depressive-like phenotype following nigrostriatal DA-ergic neurodegeneration (Drui et al., 2014). However, we could observe a significant association between immobility time in the tail suspension test and meander as measure of perseverative behavior, indicating that the decrease in immobility of the LAC-treated mice can be due to increased perseverance to engage in escape-oriented behaviors. Our results thus highlight that cognitive impairment can confound measures of depressive-like symptoms in “behavioral despair” paradigms. Due to the strong influence of perseverative behavior on non-motor phenotypes related to anxiety and depression, we believe that the intranigral LAC model is particular suited to study loss of cognitive flexibility in PD. At the same time, we cannot completely dissociate a pure anxiogenic effect of the LAC lesion that might have occurred in addition to changes in cognitive behavior.

It would be interesting to speculate on the cause of the observed non-motor behaviors, especially taking into account the partial DA-ergic lesion induced by LAC. Partial damage to the nigrostriatal DA-ergic pathway has been linked with the development of somatosensory dysfunction (Fornaguera et al., 1993), loss of cognitive flexibility (Darvas et al., 2014), and development of anxiety-like behavior (Drui et al., 2014), phenotypes which we document in our model. Furthermore, besides nigral cell loss, we observed a localized loss of VTA DA-ergic neurons situated in the PBP at the injection site. It is conceivable that even a partial loss of TH+ neurons in the VTA could have contributed, to a certain extent, to the observed non-motor behaviors. Consistent with such a view are observations indicating that partial VTA lesions can induce perseverative behavior (Pioli et al., 2008), hyperactivity (Koob et al., 1981), and contribute to sensorimotor neglect developed after a nigrostriatal lesion (Lees et al., 1985). Additionally, damage to non-DA-ergic systems following LAC could have influenced some of the observed non-motor features. Indeed, compared to other PD toxins that show specificity for DA-ergic neurons (such as 6-OHDA), the active metabolite of LAC diffuses freely through cell membranes (Dick et al., 1997), although DA-ergic neurons might be particularly sensitive to proteasome-inhibition mediated cell death (Petrucelli et al., 2002; however also see Reaney et al., 2006). In order to evaluate possible non-specific damage of the lesion on surrounding cell populations, we chose to focus on the GABA-ergic neurons of the SNr in immediate proximity of the injection site. Our findings indicate that intranigral LAC lesion did not significantly damage PV+ neurons in the SNr, supporting the notion that GABA-ergic neurons are more resistant to proteasomal dysfunction than DA-ergic neurons (Rideout et al., 2005). Although we exclude a potential influence of damage to the SNr on the observed behavior, LAC injection might have also affected other nuclei located in close proximity to the injection site (Vernon et al., 2010), or that project to the SNc such as the pedunculopontine nucleus (PPN). Indeed, it was recently demonstrated that intranigral LAC administration in rats leads to a loss of cholinergic neurons in the PPN (Pienaar et al., 2015), similar as to the loss observed in PD patients. Although not assessed in the current study, we acknowledge that damage in extra-nigral areas, such as the PPN, could have participated to the development of non-motor symptoms in our model. For instance, PPN-lesioned rats demonstrate difficulties in terminating a strongly selected action (sucrose preference), potentially highlighting an increase in perseverative behavior (Winn, 2006).

As a final characterization of our model, we evaluated asymmetry in body swings during escape-oriented behavior. Indeed, the elevated body swing test revealed that rats lesioned with 6-OHDA have increased swing bias toward the side contralateral to the lesion, an effect that is sensitive even in conditions of moderate DA depletion (Borlongan and Sanberg, 1995; Roghani et al., 2002). Nevertheless, despite one earlier report in the literature (Iancu et al., 2005), this test has not been fully evaluated in mouse models of PD. The similarities in test set-up between the tail suspension test and the elevated body swing test in mice (Iancu et al., 2005), made us wonder whether we could use the tail suspension video recordings to integrate body swing bias. Our results showed a global increase in contralateral body swings in LAC lesioned mice, confirming motor asymmetry. Furthermore, these findings indicate that the tail suspension test can be combined with the elevated body swing test for simultaneous phenotyping of motor and non-motor features in unilateral models of PD, thereby increasing the information density of the test (Gerlai, 2002), and reducing the number of animals used (in line with the “Reduction” component of the 3Rs principle, Kilkenny et al., 2010).

In conclusion, we found that intranigral administration of LAC to mice leads to nigrostriatal DA-ergic neurodegeneration and accumulation of α-synuclein and S129-P α-synuclein. LAC lesioned mice demonstrate motor impairment, as well as non-motor features resembling early stage PD, including sensitive and somatosensory alterations, anxiety-like behavior, and perseverative behavior. At the same time, we acknowledge certain limitations of the model with regard to translatability to the human disorder. First, similar to other toxin-based models of PD, the intranigral LAC model is characterized by an acute, and non-progressive neurodegenerative process, that could be different than the one occurring in a chronic neurodegenerative disorder as PD (Duty and Jenner, 2011). Second, although the preference of LAC to specifically induce DA-ergic neurodegeneration has been previously documented *in vitro* (Petrucelli et al., 2002; Mytilineou et al., 2004; Rideout et al., 2005), the indiscriminate mechanism of cell entry LAC might cause non-specific damage to neurons or glial cells, and should be carefully considered. Yet, with taking these limitations into account, the model demonstrates good construct and face validity, and represents an alternative platform to evaluate therapeutic strategies in PD, due to its different, but relevant mechanism of inducing DA neuronal loss. Furthermore, the characterization of the model in mice provides an advantage for subsequent application in transgenic mice, in order to investigate molecular determinants of proteasomal inhibition-induced neurodegeneration and validate therapeutic targets.

## Author contributions

EB, YM, IS, VB, and AM designed the experiments. EB performed surgeries, behavioral phenotyping (with JV, TD, GA, and EM), immunohistochemistry (with AV for detection of α-synuclein and S129-P α-synuclein), HPLC experiments (with AE), and data analysis (with JV, TD, GA, AV, and AE.) and interpretation. EB and AM wrote the manuscript. All authors reviewed and commented on the manuscript, and approved it in its final form.

### Conflict of interest statement

The authors declare that the research was conducted in the absence of any commercial or financial relationships that could be construed as a potential conflict of interest.

## Supplementary material

Supplementary Figure 1 **Rostro-caudal distribution of S129-P α-synuclein (A) and α-synuclein (B) increased immunoreactivity through the SN, following LAC administration**. Increased expression of S129-P α-synuclein and α-synuclein could be observed through the entire rostro-caudal extent of the SN in mice unilaterally lesioned with LAC. Scale bar 400 μm.

Supplementary Figure 2 **Baseline motor behavior in the rotarod and adhesive removal tests**. No significant differences could be observed in baseline rotarod **(A)**, and adhesive removal performance **(B)**, indicating similar acquisition of motor skilled behaviors between experimental groups. Data are presented as mean ± s.e.m., *p* > 0.05 (One-Way ANOVA). Sample size indicated in the figure.

Supplementary Video 1 **“Behavioral despair” in the tail suspension test**. After a period of initial struggle, control mice demonstrate intervals of immobile behavior, reflecting reluctance to maintain active escape-oriented behavior. Indicated is a representative 1 min interval from the tail suspension test of a sham-treated mouse (trial period: 2 min 30 s—3min 30 s), during which the mouse shows primarily immobile behavior. The video is shown at 4x its normal speed.

Supplementary Video 2 **Minor akathisia-like behavior in mice treated with 3 μg LAC**. In comparison to sham mice, LAC-treated mice demonstrate less immobile behavior and increased restlessness in the tail suspension test. Indicated is a representative 1 min interval from the tail suspension test of a LAC-treated mouse (trial period: 2 min 30 s—3 min 30 s), during which the mouse shows minor akathisia-like behavior (periods of continuous movement, combined with intervals of immobility). The video is shown at 4x its normal speed.

Supplementary Video 3 **Strong akathisia-like behavior in mice treated with 3 μg LAC**. In comparison to sham mice, LAC-treated mice demonstrate less immobile behavior and increased restlessness in the tail suspension test. Indicated is a representative 1 min interval from the tail suspension test of a LAC-treated mouse (trial period: 2min 30 s—3 min 30 s), during which the mouse shows strong akathisia-like behavior (continuous movement/restlessness). The video is shown at 4x its normal speed.
