# Supplementary figures and images for: Nigral proteasome inhibition in mice leads to motor and non-motor deficits and increased expression of Ser129 phosphorylated α-synuclein
Source: Front Behav Neurosci. 2015 Mar 31;9:68. doi: 10.3389/fnbeh.2015.00068 (PMC4379937; doi:10.3389/fnbeh.2015.00068)

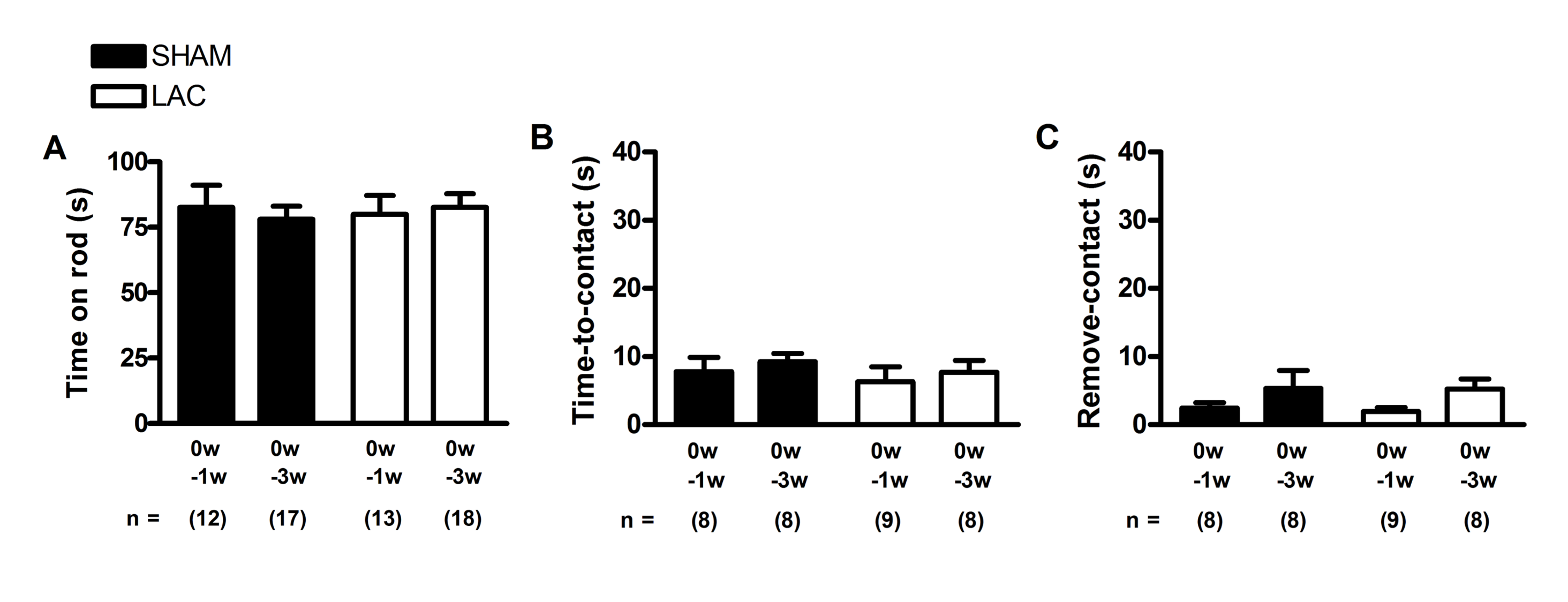

Supplement: Supplementary Figure 2 — Baseline motor behavior in the rotarod and adhesive removal tests. No significant differences could be observed in baseline rotarod (A), and adhesive removal performance (B), indicating similar acquisition of motor skilled behaviors between experimental groups. Data are presented as mean ± s.e.m., p > 0.05 (One-Way ANOVA). Sample size indicated in the figure. [file Image2.TIF]
